# Housing Risk Factors of Four Tropical Neglected Diseases: A Brief Review of the Recent Literature

**DOI:** 10.3390/tropicalmed7070143

**Published:** 2022-07-21

**Authors:** Anouk H. M. Chastonay, Oriane J. Chastonay

**Affiliations:** 1 Adrian Streich Architekten AG, 8004 Zürich, Switzerland; a.chastonay@gmail.com; 2 Réseau Fribourgeois de Santé Mentale, 1700 Fribourg, Switzerland

**Keywords:** adequate housing, human rights, neglected tropical disease, Chagas disease, dengue, leishmaniasis, lymphatic filariasis, risk factors

## Abstract

Alongside peace, education, food, income, a stable ecosystem, sustainable resources and social justice, *shelter* is a prerequisite for health. According to international human rights law, everyone is entitled to an adequate standard of living, which includes adequate housing. Adequate housing, including access to water and sanitation, plays a critical role in the prevention and management of neglected tropical diseases, which affect over 1 billion people worldwide. Inadequate housing conditions represent a risk factor for many of them, e.g., Chagas disease that affects 6–8 million people worldwide, visceral leishmaniasis that kills 20,000–30,000 people/year, lymphatic filariasis which threatens 859 million people worldwide or dengue that has increased 8–10 fold over the last two decades. Vector control strategies for the above-mentioned diseases have shown their effectiveness and should include systematic and repetitive in-house spraying and individual protection (e.g., impregnated nets), as well as better-quality construction material and techniques and better sanitation infrastructures and practices. Access to adequate housing is a basic human right. The violation of the right to adequate housing may affect the enjoyment of other human rights. Access to adequate housing can strengthen (and facilitate access to) other basic human rights, such as the rights to work, health, security, and education.

## 1. Introduction

The World Health Organization (WHO) in its *Milestones in Health Promotion* states that alongside peace, education, food, income, a stable ecosystem, sustainable resources, social justice and equity, *shelter* is a prerequisite for health [1], as also suggested in the Ottawa Charter for Health Promotion [2]. This in turn implies building healthy public policy and a sustainable environment [3].

More recently, the WHO reaffirms the importance of decent housing conditions, which *“can save lives, prevent disease, increase quality of life, reduce poverty [and] help mitigate climate change”* [4]. As for Brauchbach and Savelsberg [5], after analyzing the WHO Lares database, they concluded that inadequate housing conditions have quite an impact on health outcomes (e.g., falls, respiratory diseases, gastrointestinal diseases, depression, etc.). Indeed, poor housing, notably through indoor air pollution, inappropriate water supply as well as inadequate sanitation facilities, enhances the development of communicable diseases [4] and contributes to premature mortality (e.g., according to the *Global Burden of Disease* study 1.6 million people died prematurely in 2017 because of indoor air pollution [6]), including in industrialized countries (e.g., more than 100,000 deaths annually in Europe [7]). Furthermore, poor housing conditions increase the risk of “severe ill-health or disability by up to 25 percent during childhood and early adulthood” [8]. At last, poor housing conditions contribute to health inequalities [4].

Regarding *neglected tropical diseases (NTDs)*, Hunt, the former United Nations Special Rapporteur on the right of everyone to the enjoyment of the highest attainable standard of physical and mental health, and colleagues have insisted in their report, *Neglected diseases:* *A human rights analysis* on the links between NTDs and basic human rights, the right to adequate housing being one of the basic human rights mentioned the following [9]: *“Neglected diseases are both a cause and consequence of human rights violations. The failure to respect certain human rights, such as the rights to water, adequate housing, education and participation, increases the vulnerability of individuals and communities to neglected diseases. People afflicted by neglected diseases are vulnerable to violations of their human rights, including the rights to health, life, non-discrimination, privacy, work, education, and to enjoy the benefits of scientific progress”.*

Regarding *the Right to Adequate Housing,* according to international human rights law, everyone is entitled to an adequate standard of living, which includes adequate housing. Despite this, more than a billion people do not enjoy adequate housing conditions, living, inter alia, in health-threatening conditions [10]. Adequate housing was recognized as part of the right to an adequate standard of living in the Universal Declaration of Human Rights (1948) [11] and in the International Covenant on Economic, Social and Cultural Rights (1966) [12]. *“Adequate housing must provide more than four walls and a roof”* [10]. For housing to be adequate, it must provide *security of tenure*, *affordability, accessibility, appropriate* *location, and cultural adequacy*. Furthermore, it must provide *appropriate habitability*, i.e., it must *“guarantee physical safety or provide adequate space, as well as protection against the cold, damp, heat, rain, wind, other threats to health”* [10]. There must also be facilities essential for health, such as safe drinking water, heating, lighting, energy for cooking, sanitation and washing facilities, means of storing food, refuse disposal, site drainage [13] as well as protection from threats to health such as structural hazards and disease vectors [10].

Adequate housing, including access to water and sanitation, plays a critical role in the prevention of diseases and their management, including NTDs, which affect over 1 billion people worldwide [4], even though mass drug administration programs, for several NTDs, have been quite successful, despite its logistic difficulties, according to field data [14] and to the opinion of experts [15].

We decided to review the recent literature having investigated housing risk factors of four vector borne NTDs, which all together threaten/affect several hundred million people worldwide annually, our hypothesis being that recent studies still show that there are important risk factors related to housing conditions, risk factors that could/should be tackled to reduce the burden of those diseases as well as in a human rights perspective. The four NTDs considered here are as follows:

*Chagas Disease (CD):* Chagas disease is an anthropo-zoonosis disease caused by the protozoan parasite *Trypanosoma cruzi*, mainly transmitted by hemiptera insects, known as the kissing bugs [16]. It affects 6 to 8 million people worldwide. It causes an estimated 50,000 deaths per year. Around 65–100 million people live in areas at risk for infection worldwide. In 21 countries of the Americas, the disease is endemic, affecting approximately 6 million people with an annual incidence of 30,000 new cases and 12,000 deaths [17].

*Leishmaniasis (L):* Leishmaniasis is caused by *protozoa parasites*, which are transmitted to humans by the bite of infected female *phlebotomine sandflies*, resulting in the following three main forms of the disease: *cutaneous leishmaniasis* (CL), *visceral leishmaniasis* (VL) and mucocutaneous leishmaniasis. Clinical manifestations range from skin ulcers to lethal systemic disease. An estimated 700,000 to 1 million new cases occur each year worldwide. CL represents way over 600,000 cases, which mainly occur (95%) in the Americas, the Mediterranean Basin, the Middle East, and Central Asia. VL represents between 50,000 and 90,000 new cases/year. Most cases of VL occur in Brazil, East Africa and India. VL is a fatal disease in 95% of the cases if left untreated (presently an estimated 20,000 to 30,000 deaths occur annually) [18].

*Lymphatic Filariasis (LF):* Lymphatic filariasis is *caused by a parasite (Wuchereria bancrofti,* responsible for 90% of the cases) and transmitted by different types of mosquitoes (*Culex, Anopheles, *Aedes)**. It can lead to elephantiasis, causing pain, severe disability and social stigma. According to WHO, *“51 million people were infected as of 2018, a 74% decline since the start of WHO’s Global Program to Eliminate Lymphatic Filariasis in 2000”. Yet “859 million people in 50 countries worldwide remain threatened by lymphatic filariasis and require preventive chemotherapy to stop the spread of this parasitic infection”* [19,20]. One-third of people infected live in Africa, another third in India and the remainder in the Americas, the Pacific Islands and Southeast Asia.

*Dengue (D):* Dengue is a vector-borne viral infection (*Dengue Virus*) affecting an estimated 100–400 million people each year. It is transmitted to humans through the bite of infected mosquitoes (primary *Aedes aegypti* mosquitoes). Dengue occurs mostly in urban and semi-urban areas of tropical and sub-tropical regions. Up to 80% of infections are mild or asymptomatic. Yet, severe dengue is a leading cause of death in some countries in Latin America and Asia, which bear up to 70% of the burden of the disease. The global incidence of dengue has grown over the past decades, with about half of the world’s population now at risk. Dengue’s impact today is 30 times greater than it was just 50 years ago [21,22].

## 2. Methods of the Literature Review

*Research strategy:* We limited the research to the PubMed database over a 10-year period (2012–2021) centered on freely available articles in English. The research strategy was formulated as follows: “housing conditions” AND “risk factors” AND (Chagas disease OR Leishmaniasis OR Lymphatic Filariasis OR Dengue).

*Exclusion criteria:* Articles centered on interventions, not risk factors. Articles presenting no numerical data on housing conditions. Articles not specifically centered on one of the four diseases.

*Analyzing process:* Data presented in each included article were analyzed regarding evidence of any confirmed or not confirmed housing risk factors specifically mentioned (mud walls/floors, thatched houses/roofs, sanitation, clean water) and statistical information (OR) were extracted.

**Figure 1:**
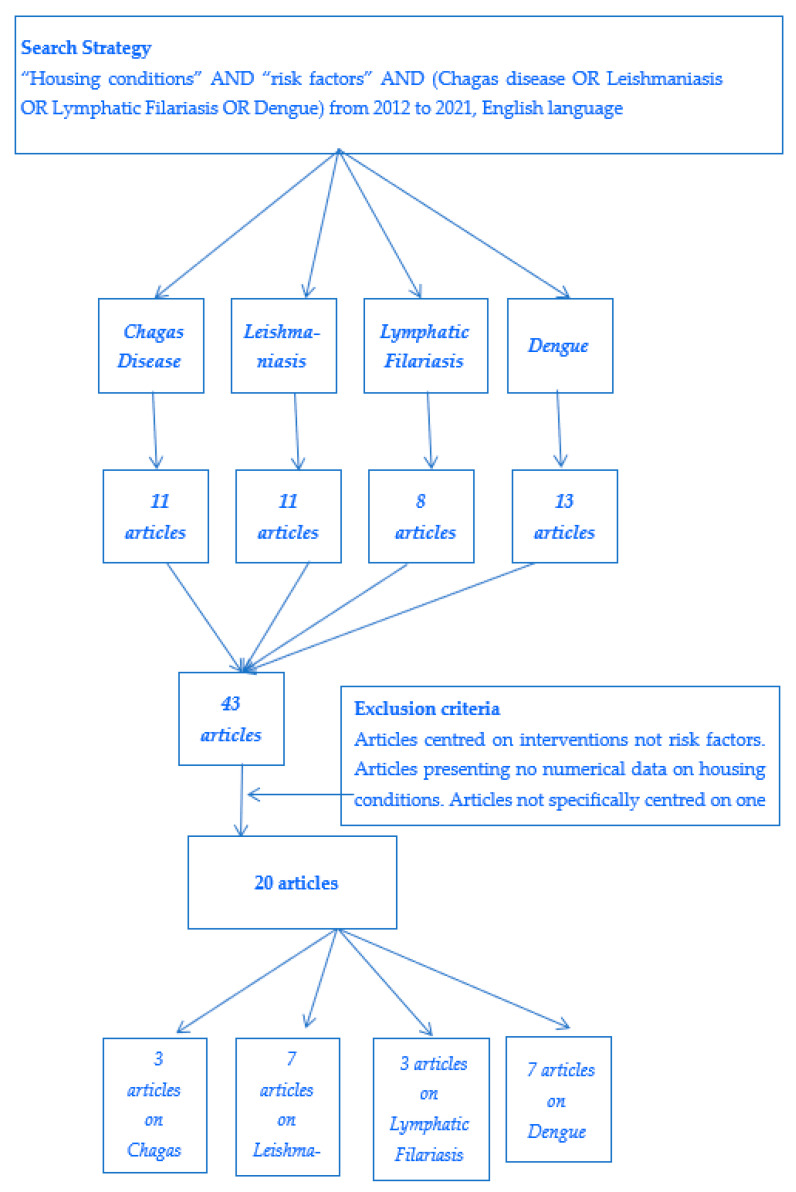
Research strategy (limited to PubMed) and results.

The authors performed the literature research together. The process is summarized in Figure 1.

## 3. Results of the Literature Review concerning Housing Risk Factors of Four Tropical Neglected Diseases

The adopted research strategy yielded, over the 10-year period considered, 11 articles related to Chagas disease, 11 to leishmaniasis, 8 to lymphatic filariasis and 13 to dengue (list annexed). In the analysis process, studies presenting original numerical data of risk factors were included, i.e., 3/11 articles related to CD, 7/11 to L, 3/8 to LF and 7/13 to D. The results are summarized in Table 1.

### 3.1. Chagas Disease

The main observations from the three articles included are summed up hereafter.

Bustamante et al. [23] in their cross-sectional survey on risk factors associated with persistent domiciliary *Triatoma dimidiata* infestation in two regions in Guatemala report, regarding construction conditions, as follows:

- Walls containing *bajareque* (mud and sticks), but not adobe, were a risk factor (OR = 1.9, 95% CI 1.2–3.9) as well as earthen floors (OR = 3.4, 95% CI 1.9–6.0) and tile roofs (OR = 1.9, 95% CI 1.1–3.3); on the contrary, cement or tile floors were protective (OR = 0.3, 95% CI 0.2–0.7) as well as cinder block walls.

In parallel, these authors report that high domiciliary dog density and in-house rodent presence increase the infestation risk. They conclude that integrated vector control strategies should be considered.

Crocco et al. [24] in their cross-sectional survey on risk factors associated with the presence of *triatomines* in rural areas of south Argentina report the following:

- Houses with unplastered walls had a 20.7 times greater risk of infestation (*p* < 0.001) than those with uncracked plastered walls; similarly houses with unplastered concrete or brick, or thatched roofs had a 7.2 times greater risk of infestation than houses with plastered roofs (*p* < 0.001).

These authors also report infestation indices of 59.7% for house compounds and 58.3% for peridomestic areas, underlining the importance of raising the awareness of inhabitants of the peridomiciliary environment, which must be improved through vector control strategies.

Lardeux et al. [25] in the context of their experimental study on controlling *Triatoma infestans* in four poor rural villages of Bolivia report that as risk factors for the presence of *Triatoma infestans,* bad wall conditions (defined as walls with numerous cracks and crevices) (Chaco region: OR = 3.9, 95% CI 2.3–6.7; Valleys region: OR = 4.4, 95% CI 0.94–18.2); whereas, thatched roofs were an established risk factor in three villages out of four (OR = 1.5, 95% CI 0.2–13.3; OR = 1.0, 95% CI 0.3–3.3; OR = 3.7, 95% CI 1.6–8.7). The presence of chickens in the house was a further risk factor. In parallel to their study, the authors-initiated community vector control activities (coating walls, house cleaning activities and removal of in-house animals’ activities, which yielded a notable decrease in *Triatoma infestans* infestation.

### 3.2. Leishmaniasis

The main observations from the articles included are summed-up hereafter.

Younis et al. [26] in a case-control study from Nepal (plain and hilly area) published in 2020 analyzed housing structures and land lot data from 66 VL and 137 controls, exploring possible risk factors of VL. Risk factors with the highest odds of VL concerning housing were bamboo walls (AOR = 8.1, 95% CI 2.4–27.6), cracks in bedroom walls (AOR = 2.9, 95% CI 0.9–9.2) and sacks near sleeping areas (AOR = 19.2, 95% CI 4.06–90.46). Furthermore, several studies mention that clinically diagnosed VL was statistically significantly associated with thatched houses without windows compared to thatched houses without windows (urban Nepal): (AOR = 0.4, 95% CI 0.1–0.8) [27], cracked house walls (semi-urban Ethiopia): (AOR = 6.4, 95% CI 1.6–25.6) [28], thatched and/or mud house (rural India): (OR = 6.6; 95% CI 1.8–23.7) [29]. The authors recommend, inter alia, elimination and educational programs should include housing improvement.

Concerning CL, several studies report precarious housing conditions besides poverty as risk factors, e.g., as follows:

- Houses without water supply (AOR = 6.0, 95% CI 2.7–13.1) in a study from an endemic area in Brazil [30].
- Poor interior housing conditions (OR = 2.0, 95% CI 1.0–3.93), among other major risk factors such as chronic diseases in a study from south-eastern Iran [31].
- Non-plastered brick walls (OR = 41.5, 95% CI 13.8–124.8) in a study from southern Sri Lanka [32].

These authors plead for improving house construction, water supply and sanitation as well as protecting high risk individuals as well as vector control measures and public education regarding prevention.

### 3.3. Lymphatic Filariasis

Studies have investigated the socioenvironmental conditions—including inadequate housing conditions—as a risk factor for transmission of lymphatic filariasis, e.g., as follows:

Mutheneni et al. [33] in their case-control study on the influence of socioeconomic aspects on lymphatic filariasis in Andhra Pradesh, India showed, regarding housing conditions, that the risk of filariasis was higher in groups of people living in tiled house structures (OR = 1.6, 95% CI 0.5–5.0) with a kutcha (uncemented) drainage system (OR = 19.4, 95% CI 3.0–126.4). The authors of their study also reported that the population with low and medium socioeconomic status is at higher risk of filariasis than those not aware of prevention measures. They recommend integrating those various aspects into the prevention and management of filariasis.

Srividya et al. [34] in their study implemented in Tamil Nadu state, India, where the population had undergone eight annual rounds of mass drugs identified among 33 sites, 12 hotspots (Microfiliaria prevalence > 1% or Ag positive children in the age group of 2–8 years). Logistic regression revealed that tiled and concrete houses increased the risk of an area being a hotspot by 2.0 and 2.9 times, respectively. The presence of Culex breeding habitats was significantly associated with an elevated risk of being a hotspot. The proximity of U-drains to a house increased the risk of filarial infection 5.8 times. The authors suggest that those residual risk factors may be potential resurgence/transmission foci.

Upadhyayula et al. [35] in a cohort study of lymphatic filariasis related to socio-economic conditions in Andhra Pradesh, India, found that house structure (hut OR = 1.9, 95% CI = 1.2–3.1; tiled OR = 1.3, 95% CI = 0.8–2.0) was found to be highly associated with the occurrence of filarial disease. Indeed, thatched/tiled houses showed a significant increase in infections when compared to reinforced cement concrete structures (*p* = 0.032) as well as in housing with kutcha drainage (mud drainage system) compared to pucca drainage (masonry drainage system) (*p* = 0.001). Other socioeconomic variables such as lower educational status (OR = 2.6, 95% CI = 1.1–6.5) were also associated with higher infection rates.

### 3.4. Dengue

A study by Lippi et al. [36] from a dengue hyper-endemic city (Machala) in Ecuador showed that Aedes aegypti presence was associated inter alia with interruptions in water service (OR = 1.7, 95% CI 1.10–2.55), whereas the existence of air-conditioning diminished the presence of Aedes aegypti. When comparing households with dengue infection to households without, shaded patios were a significant risk factor (OR = 3.8, 95% CI 1.3–11.5), whereas window screens in good condition were a protective factor (OR = 0.3, 95% CI 0.1–0.8). Lippi et al. [37] in another study from Guayaquil (another Ecuadorian dengue hyper-endemic city), reported that an important factor associated with the presence of dengue cases was poor housing conditions (e.g., the poor structural condition of the floor, roof and walls) (OR = 24.6, 95% CI 17.6–32.1).

Rahman et al. [38] in a study on ecological, social and environmental determinants of dengue vector abundance in urban and rural north-eastern Thailand reported that when considering a Premise Condition Index (an index integrating house conditions, yard conditions, shade conditions, water supply and storage), the abundance of Aedes aegypti (female adults) was highly correlated with a high index (IRR = 2.0, 95% CI 1.5–2.6), i.e., bad house conditions, untidy yard, shady condition of house and yard (>50%), rainwater and/or open water source. For the authors, the identification of those risk factors is important for effective vector control and disease prevention.

Martin et al. [39] investigated socio-ecological factors associated with Aedes aegypti in Huaquillas, Ecuador and reported that homes were more likely to have Aedes aegypti when households had interruptions in piped water service (OR = 4.8, 95% CI 1.1–24.1) and less likely when households had septic tank systems (OR = 0.1, 95% CI 0.01–0.1). Based on their findings, these authors mentioned that infrastructure access is important for vector control.

In a study in an urban community in Nepal, Shah et al. [40] reported several risk factors for dengue transmission, i.e., water storage in an open tank in the household was associated with transmission of dengue virus (AOR = 3.8, 95% CI 1.5–9.4); a collection of dirty water around houses favored breeding sites for mosquitoes (OR = 1.9, 95% CI 1.0-3.7). Furthermore, water collected in discarded containers/tires was associated with a six-times stronger risk of dengue transmission (AOR = 6.3, 95% CI 2.7–14.5).

Regarding housing conditions, the mentioned researchers consider that screening houses for vectors, improving construction techniques and housing conditions, improving access to clean/piped water as well as promoting personal protective measures should be enhanced.

Table 1 presents the data in a condensed way.

## 4. Discussion

First, let us emphasize that our review suffers from the following several limitations:

- It is limited to a rather short period of time (10 years), thus excluding possible major studies going back to previous decades.
- Only studies listed on a single platform were considered (PubMed), which may have contributed to missing meaningful studies, since various platforms may search additional databases.
- Articles exclusively in the English language were considered, which might have excluded critical studies published in other languages, even more since the discussed diseases are mostly endemic in non-English-speaking countries.

Bad housing conditions as a risk factor for many neglected tropical diseases have been recognized early on, which triggered many, often successful, intervention programs aimed at improving housing conditions, be it through spraying measures against vectors, improving access to tap water, developing sewage disposal systems or drastic sanitation measures in buildings and neighborhoods.

Concerning the four diseases discussed in the present paper, housing conditions as potential risk factors have also been identified for many decades, as follows: let us mention examples regarding the following:

- Chagas disease: In the early 1990s, Starr et al. [41] reported from Costa Rica higher relative odds of *Triatoma dimidiate* infestation when the floor type was dirt versus other types of floor (OR = 1.7, 95% CI 0.8–3.8); or the wall type was earthen versus other types of wall (OR = 1.6, 95% CI 0.8–2.9); or the roof was made of tiles versus galvanized metal roof (OR = 2.4, 95% CI 1.1–5.4);
- Leishmania: In the early 1990s, Weigel et al. [42] reported environmental risk factors of CL in Columbia, among which they mentioned *roof thatch or palm leaves* (OR = 2.0, 95% CI 1.3–3.2). More recently, Singh et al. [43] reported from India that housing conditions represent risk factors for VL independently of socioeconomic status *Living in a thatched house* (OR 2.60, 95% CI 1.50–4.48) or *Living in a house with damp floors* (OR 2.6, 95% CI 1.25–5.41);
- Lymphatic filiaria: In the late 1970s, Maheudin et al. [44] reported from Indonesia that persons living in poorly built houses had a nine times higher microfila infection rate and a five times higher disease rate than people living in modern houses.
- Dengue: In the late 1980s, Focks and Chadee [45], recognizing the need to control *Aedes aegypti* proliferation, estimated in the context of Trinidad that the provision of an adequate water supply system and an environmental sanitation effort would eliminate the ubiquitous small water containers (buckets, tires, etc.) would reduce mosquito densities by >80%.
- The international community has long recognized the importance of improving housing conditions to prevent/control/eradicate, among others, the above-discussed diseases. Indeed, many projects have been initiated and implemented worldwide based on guidelines/recommendations/roadmaps, such as the following:
- *Keeping the vector out: housing improvements for vector control and sustainable development* [46], which argues based on evidence that poor quality housing and neglected peridomestic environments are risk factors for the transmission of many diseases including, Chagas disease, leishmaniasis, filariasis and dengue, and those housing interventions such as screening windows, reducing cracks in walls, floors and roofs are essential.
- *WASH (Water, Sanitation and Hygiene)* [47]: Safe drinking water, sanitation and hygiene are crucial to human health. Evidence suggests that clean drinking water (piped water) and connections to sewer systems will improve health outcomes, notably mortality related to diarrhea. Regarding NTDs, one of five key strategies identified to combat those diseases is “*safe drinking-water, basic sanitation and hygiene services*”, which can contribute to reducing the incidence and morbidity of water-associated vector-borne diseases such as dengue or filariasis or, through closed sewerage systems, decrease breeding and resting sites of vectors, such as sand-flies transmitting leishmaniasis [48];
- *WHO NTDs Roadmaps* [49]: Regarding the four NTDs discussed here, the roadmap for neglected tropical diseases 2021–2030 [49] proposes, concerning risk factors due to housing conditions, core strategic interventions, such as sanitation improvements that can reduce vector breeding habitats, insecticide spraying, insecticide-treated nets and environmental management, reducing available habitats for mosquitoes (e.g., environmental modification, house construction), home cleanliness and housing improvements (e.g., crack-free walls, bed-nets).

Data presented here suggest that the vector control strategies should include better quality construction materials and techniques as well as better sanitation infrastructures and practices. Especially systematic and repetitive in-house spraying and individual protection (e.g., impregnated nets) are recommended.

Indeed, one should keep the following in mind:

- Mud walls are more prone to cracks/holes that facilitate breeding sites for potential vectors. Mud walls can also retain moisture for prolonged periods, ensuring optimal humidity corresponding to a protective environment for vectors, thus potentially increasing the density of vectors; for example, shown with *Culex* [50,51].
- Similarly, houses with damp floors or thatched houses could provide an adequate environment for the survival of potential vectors [29,43,44]. An inadequate sewage system, as well as backyard characteristics (swamp, for example), may also play a role in offering favorable breeding/surviving conditions to potential vectors [52].

Yet, hard-core evidence (established through randomized controlled trials) is scarce that improved construction techniques and construction materials do radically change the health outcomes of the people concerned regarding the above-mentioned diseases. This seems in part related to a lack of random controlled interventions reported in the scientific literature, as suggested by Horstick and Runge-Ranzinger in their systematic review of “*Vector control interventions providing protection against Chagas disease, dengue, leishmaniasis, and lymphatic filariasis at the household level*” [53]. These authors have assessed 1416 articles, eventually including 32 articles considered of good quality in their review, i.e., randomized controlled trials (RCT) and cluster randomized controlled trials (cRTC). They report that the most effective interventions were intra-domiciliary spraying, insecticide-treated nets and curtains and biologically and chemically treated larval habitats. Other interventions were less effective such as waste management and clean-up campaigns to reduce the vector population. No systematic global impact on vector control was evident with modifications of the structure of homes, although they report some mixed/positive results in some specific studies on dengue and leishmaniasis, e.g., the following:

- Lime plastering (but not mud plastering) of walls significantly reduced (42%) the density of sand-flies (vector of VL) in India and one site in Nepal (but not a second one) in a study combining interventions [54];
- Environmental clean-up campaigns such as waste management indoor and outdoor as well as emptying, scrubbing, covering water containers showed a reduction of dengue illness of 24.7% and a relative risk reduction among children of dengue infection of 29.5% in a study from Nicaragua and Mexico [55].

Interestingly, Tusting et al. [56], in their systematic review and meta-analysis (conducted to assess whether modern housing is associated with a lower risk of malaria than traditional housing in malaria-endemic settings, conclude that “despite low quality evidence, the direction and consistency of effects indicate that housing is an important risk factor for malaria”. Below are the summed-up results of the 90 studies included out of 15,526 studies screened. Residents of modern houses had the following:

- 47% lower odds of malaria infection compared to traditional houses (AOR = 0.5; CI 95%: 0.42–0.67; *p* < 0.001);
- 45–65% lower odds of clinical malaria (case-control studies: AOR = 0.4, 95% CI 0.20–0.62, *p* < 0.001; cohort studies: AOR = 0.6, 95% CI 0.36–0.84, *p* = 0.005).

In another study on malaria and housing Tusting et al. [57], in their analysis of 15 Demographic and Health Surveys and 14 Malaria Indicator Surveys from 21 countries that measured malaria infection, reported that “across all surveys modern housing was associated with a 9% to 14% reduction in the odds of malaria infection” (AOR = 0.9, 95% CI 0.85–0.97, *p* = 0.003 with microscopic diagnosis; AOR = 0.9, 95% CI 0.80–0.92, *p* < 0.001 with rapid diagnostic test).

Even though indoor control of vector density based on systematic spraying seems to be the most effective intervention regarding the above-mentioned diseases, there needs to be a long-term strategy that includes housing improvement as suggested by various researchers and international organizations [4,58,59]. Indeed, housing structures can have an impact on the overall health, be it physical or mental, of their inhabitants [4,46]. Furthermore, housing structures may affect the socio-economic conditions of their inhabitants, which in turn impacts their health [5,60]. Such considerations gave rise to projects such as the *Healthy Homes for Healthy Living Model,* which proposes *“a strategy focused on building and promoting living environments designed to deter presence of vectors in domestic and peridomestic areas”* through the structural improvement of homes, health promotion activities and community involvement [59]. However, the structural improvement of homes may face logistic challenges, such as access to appropriate technical expertise, lack of construction materials and high costs that could compromise the efficacy of this approach [59]. Nevertheless, there seems to be a growing interest in the potential of housing improvement as a key tool for successfully tackling vector-borne diseases such as the ones described above [61], even though important progress has been achieved through mass drug administration programs and vector control/eradication projects.

## 5. Conclusions

Access to adequate housing is a basic human right. Yet, adequate housing is more than just four walls and a roof. It must meet criteria such as security, availability of services, affordability, accessibility, and cultural adequacy. Habitability is another key criterion and especially relevant when considering the above-mentioned diseases and NTDs at large: indeed, habitability guarantees protection against the cold, damp, heat, rain, wind, other threats to health, such as vector-borne diseases and structural hazards. Thus, improved housing conditions can prevent disease, increase well-being, save lives and even reduce poverty. The support of construction professionals is therefore key.

Furthermore, since human rights are interdependent and indivisible, the violation of the right to adequate housing may affect the enjoyment of other human rights. On the contrary, access to adequate housing can strengthen (and facilitate access to) other basic human rights, such as the rights to work, health, security and education.

## Figures and Tables

**Table 1:** Housing conditions as risk/protective factors for the considered NTDs (OR-odds ratio; AOR-adjusted odds ratio; CI-confidence interval at 95%; RR-relative risk; IRR-incidence rate ratio; *p*-*p* value).

		Chagas Disease			Viscerla Leishmaniasis				Cutaneous Leishmaniasis			Lymphatic Filariasis			Dengue				
		Bustamante et al. [23] 2014	Croco et al. [24] 2019	Lardeux et al. [25] 2015	Younis et al. [26] 2020	Uranw et al. [27] 2013	Yared et al. [28] 2014	Perry et al. [29] 2013	Araujo et al. [30] 2016	Bamorovat et al. [31] 2018	Kariyawasam et al. [32] 2015	Mutheneni et al. [33] 2016	Srividya et al. [34] 2018	Upadhyayula et al. [35] 2012	Lippi et al. [36] 2021	Lippi et al. [37] 2018	Rahman et al. [38] 2021	Martin et al. [39] 2021	Shah et al. [40] 2021
Risk factor House	Poor housing conditions									OR 2.0 CI 1.0–3.9						OR 24.6 CI 17.6–32.1	IRR 2.0 CI 1.5–2.6		
	Thatched/mud/hut house		RR 7.2 *p* < 0.001					OR 6.6 CI 1.8–23.7						OR 1.9 CI = 1.2–3.1					
	Unplastered concrete/brick/tiles walls		RR 20.7 *p* < 0.001								OR 41.5 CI 13.8–124.8		RR 2.0 RR 2.9						
	Bajareque walls (Mud and sticks)	OR 1.9 CI 1.2–3.9																	
	Bamboo walls				AOR 8.1 CI 2.4–27.6														
	Earthen floors	OR 3.4 CI 1.9–6.0																	
	Cracks in walls			OR 3.9, CI 2.3–6.7	AOR2.9 CI 0.9–9.2		AOR 6.4 CI 1.6–25.6												
	Unplastered roofs (tiles/thatched)	OR 1.9 CI 1.1–3.3		OR = 3.7, CI 1.6–8.7								OR 1.6, CI 0.5–5.0		OR 1.3 CI = 0.8–2.0					
Risk factor Water	Houses without or with interrupted water supply								AOR 6.0 CI 2.7–13.1						OR 1.7 CI 1.10–2.5			OR4.8 CI 1.1–24.1	
	Water storage in an open tank in the household																		OR0.1 CI 0.01–0.1
	Collection of dirty water around the house																		OR1.9 CI 1.0–3.7
	Water collected in discarded containers/tires																		AOR6.3 CI 2.7–14.5
	Kutcha drainage (uncemented)											OR 19.4 CI 3.0–126.4		*p* = 0.032					
	Proximity of U-drains to house												RR 5.8						
	Rainwater and open water source																		
Risk Factor Yard	Shady condition of yard and house														OR 3.8 CI 1.3–11.5		IRR 2.0 CI 1.5–2.6		
	Untidy yard																		
Protective Factor	Cement/tile floors	OR 0.3 CI 0.2–0.7																	
	Window screens in good conditions														OR 0.3 CI 0.1–0.8				
	Window vs. no window in thatched house					AOR 0.4 CI 0.1–0.8													
	Existence of air conditioning														*p* < 0.05				
	Sceptic tank system																	OR 0.1 CI 0.01–0.1	
	Pucca drainage (masonry system													*p* = 0.001					

## Data Availability

Not applicable.
